# Factors predicting renal impairment associated with Tenofovir Disoproxil fumarate: Focus on patients with HIV and pre-existing metabolic abnormalities under the Thai National Health Security Scheme

**DOI:** 10.1016/j.pmedr.2025.103154

**Published:** 2025-06-27

**Authors:** Duangjai Duangrithi, Kwandaw Silathong

**Affiliations:** a Department of Pharmacy practice, College of Pharmacy, Rangsit University, Pathumtani, Thailand; b Department of Pharmacy, Rajavithi Hospital, Bangkok, Thailand

**Keywords:** Tenofovir disoproxil fumarate, HAART therapy, Renal impairment, Metabolic abnormalities, HIV, eGFR

## Abstract

**Objective:**

The incidence of nephrotoxicity is reported as 20 % among Thai patients with HIV receiving tenofovir disoproxil fumarate (TDF). It can occur as early as 24 weeks after initiating TDF therapy. Metabolic abnormalities are the major comorbidities in patients with HIV receiving antiretrovirals. However, close monitoring for renal function and metabolic abnormalities is not possible under Thai National Health Security Scheme for either HIV or AIDS patients due to the significant budget constraints. This study aims to investigate the predictive factors of TDF associated with renal impairment in patients with HIV and pre-existing metabolic abnormalities.

**Methods:**

This longitudinal study was conducted at a university hospital in Bangkok, Thailand from January 2015 to December 2018. All participants were followed until the first occurrence of an estimated glomerular filtration rate (eGFR) ≤ 90 mL/min/1.73 m^2^.

**Results:**

Among 315 patients with pre-existing metabolic abnormalities, elevated low-density lipoprotein was the most common (47.3 %) while TDF/emtricitabine/efavirenz was the most common (75.2 %) regimen of highly active antiretroviral therapy. In addition, the body mass index (BMI) < 18.5 kg/m^2^ (odds ratio [OR] = 9.20, 95 % confidence interval [CI]: 1.11, 76.50) and baseline eGFR (OR = 0.92, 95 % CI: 0.89, 0.94) were predictive factors of renal impairment.

**Conclusions:**

For patients with HIV and pre-existing metabolic abnormalities in resource limited settings, maintaining a normal BMI and adjusting antiretroviral treatment dose according to renal function are recommended, especially in those with abnormal baseline eGFR.

## Highlights

• In HIV, metabolic abnormalities are important in resource-limited settings.
• The glomerular filtration rate started to decline after 12 weeks of treatment.
• The median onset of renal impairment was 43 weeks after starting tenofovir.
• Underweight and baseline glomerular filtration rate were the predicting factors.
• Body weight is a feasible marker for renal risk in resource-limited settings.

## 1 Background

HIV infection remains a major global public health problem. In 2022, 86 % of people living with HIV/AIDS (PLHIV) were diagnosed, and 89 % received antiretroviral therapy (ART) (HIV and AIDS, Fact sheet, 2025). These figures are projected to reach 95 % by 2025 (HIV and AIDS, Fact sheet, 2025). In Thailand, the HIV prevalence among individuals aged 15 to 49 years was 1.1 % in 2022, and 81 % of those aged ≥15 years received ART (Country factsheets, THAILAND 2022, HIV and AIDS Estimates, 2025). The annual number of new HIV diagnoses in Thailand was stable at approximately 6480 cases between 2016 and 2021 but increased to 9230 in 2022 (Division of AIDS and STDs. Annual report, 2022). This dramatic increase places a financial burden on the Thai healthcare system. In Thailand, although all patients with HIV have access to ART, this does not guarantee access to other healthcare services, such as laboratory investigations. The management of HIV/AIDS involves the use of multiple antiretroviral agents with distinct mechanisms of action to prevent HIV-1 resistance, a strategy known as highly active antiretroviral therapy (HAART). This approach has been the standard of care for HIV since 1996. HAART has significantly increased the lifespan of PLHIV. This treatment regimen typically comprises a combination of three or more antiretrovirals. Tenofovir disoproxil fumarate (TDF)-based regimen has been recommended as the first-line ART for adults according to the 2016 Consolidated Guidelines on the Use of Antiretroviral Drugs for Treating and Preventing HIV Infection (Consolidated Guidelines on HIV Prevention, Testing, Treatment, Service Delivery and Monitoring: Recommendations for a Public Health Approach, 2021). The Thailand National Guidelines on HIV/AIDS Treatment and Prevention 2017 recommend three antiretrovirals: TDF (a nucleoside reverse transcriptase inhibitor [NRTI]) combined with either lamivudine (3TC) or emtricitabine (FTC), and dolutegravir. This recommendation was adopted from the WHO guidelines in 2021 (Ruxrungtham et al., 2022). The alternative treatment includes NRTIs (TDF or tenofovir alafenamide fumarate [TAF]) plus a non-nucleoside reverse transcriptase inhibitor (NNRTI; 3TC or FTC), and efavirenz (EFV) or rilpivirine (Ruxrungtham et al., 2022). Generally, TDF has a good safety profile and is a widely used antiretroviral drug worldwide. However, an association between renal toxicity including glomerular dysfunction and proximal tubular dysfunction and TDF use has been reported. The global incidence of nephrotoxicity ranges from 0.5 to 45 % (Asirvatham et al., 2024) and from 12 % to 20 % in Thai patients with HIV (Phuphuakrat et al., 2022; Pichpattana et al., 2016; Srisopa et al., 2023). These renal adverse effects can occur as early as 24 weeks of TDF use (Asirvatham et al., 2024) and as late as 52 weeks in Thai patients treated with TDF (Suwan and Kornjirakasemsarn, 2020). The overall prevalence of renal impairment was 39 % (Calza, 2012). Its risk factors were reported as follows: female gender, older age, and low body weight or low body mass index, underlying renal dysfunction, concomitant use of nephrotoxic drugs especially, ritonavir-boosted protease inhibitors and didanosine, advanced HIV disease, and metabolic abnormalities such as hypertension and diabetes mellitus (Calza, 2012). However, studies in Thailand show conflicting results. Some studies have found no factors associated with renal dysfunction (Bunpeth et al., 2017; Pichpattana et al., 2016) while the concomitant use of protease inhibitors, hyperlipidemia, body mass index (BMI), older age, smoking and TDF use for more than three years have been associated with renal impairment (Phuphuakrat et al., 2022; Srisopa et al., 2023). Furthermore, receiving trimethoprim–sulfamethoxazole or nonsteroidal anti-inflammatory drugs, and older age were associated with proximal renal tubulopathy (Srisopa et al., 2023).

Metabolic abnormalities have become the major comorbidities among PLHIV. Metabolic syndrome was commonly found among HIV-1 infected Thai adults (Jantarapakde et al., 2014). Age ≥ 35 years was identified as a significant risk factor for metabolic syndrome (Jantarapakde et al., 2014), and the prevalence of metabolic abnormalities was found in 25–30 % of middle-aged patients with HIV receiving ART (Duangrithi et al., 2020; Jantarapakde et al., 2014). Hypertension and diabetes mellitus are the most common metabolic abnormalities in patients with HIV, especially in older individuals (Asirvatham et al., 2024). So far, several studies have been conducted on the association between HIV and metabolic abnormalities in Thailand. HIV can cause metabolic abnormalities by itself, particularly in patients with low CD4 T-lymphocyte levels, since the intestinal microbiome could be altered (Ergin et al., 2020). Consequently, systemic inflammation leading to altered metabolism may be encountered (Ergin et al., 2020). Also, impaired biogenic amine levels found in patients with untreated HIV may be related to metabolic dysregulation (Ergin et al., 2020). Females, older adults, and patients with high BMI were at a higher risk of metabolic abnormalities (Ergin et al., 2020). Unfortunately, monitoring nephrotoxicity from TDF is not possible under the National Health Security Scheme for Thai patients with HIV due to limited resources. Only serum creatinine and urinalysis are allowed before treatment initiation and every six months in patients treated with TDF-based regimens. Fasting plasma glucose and lipid profile testing can be performed twice a year in patients >35 years of age (Ruxrungtham et al., 2022). Correspondingly, The Thai National Health Security Scheme for patients with HIV covers the expense of renal function test, fasting plasma glucose and lipid profile testing twice a year. To maximize patient care in resource-limited settings, early detection of renal impairment may serve as a valuable indicator of TDF-induced nephrotoxicity. In the light of these considerations, this study aimed to investigate the predictive factors of renal impairment in patients with HIV and pre-existing metabolic abnormalities.

## 2 Methods

### 2.1 Setting and policy

This longitudinal study was conducted at a university hospital in Bangkok, Thailand in accordance with the Declaration of Helsinki. Ethics approval was granted by the Rajavithi University Hospital review board on January 16, 2020. The approval number is 63016. Patients registered at HIV clinics from January 2015 until December 2018 were invited to participate in the study and were provided with a written informed consent.

The Thai National Health Security Scheme, nonetheless, faces financial limitations in supporting patients with HIV and AIDS, influencing the Thailand National Guidelines on HIV/AIDS Treatment and Prevention. The recommended laboratory investigations consisting of complete blood count, fasting blood sugar, creatinine, cholesterol, triglyceride, alanine transaminase (ALT), viral load, and CD4 were restricted to twice a year. Drug resistance testing was permitted annually while anti-hepatitis C virus (HCV) and anti-hepatitis B surface antigen testing were confined to a single point during the treatment course.

### 2.2 Participants

Patients aged >18 years old, receiving HAART regimens containing tenofovir for at least one year, with estimated glomerular filtration rate (eGFR) > 90 mL/min/1.73 m^2^ and having at least one metabolic abnormality were eligible. Patients with a history of HAART containing tenofovir, kidney disease, or renal impairment were excluded. Laboratory testing was performed according to the Thailand National Guidelines on HIV/AIDS Treatment and Prevention (Department of Disease Control, Thailand National guidelines on HIV/AIDS treatment and prevention, 2017), as well as the Thai National Health Security Scheme for patients with HIV. Apart from the required laboratory testing, all participants were monitored for renal function at each visit until the first episode of eGFR ≤90 mL/min/1.73 m^2^.

The incidence of TDF-induced nephrotoxicity ranged from 0.5 to 45 % among PLHIV (Asirvatham et al., 2024). Based on this, the incidence of renal dysfunction was estimated at 23 % with a 95 % confidence level, a 5 % margin of error, and a 15 % loss rate. Therefore, the sample size of at least 315 participants was required for this study.

### 2.3 Definitions

The pre-existing metabolic abnormalities were defined as at least one of the following: blood pressure over 130/85 mmHg or hypertension, fasting triglyceride level over 150 mg/dL or high-density lipoprotein less than 40 mg/dL in males and 50 mg/dL in females or dyslipidemia, fasting blood sugar over 100 mg/dL or diabetes mellitus and BMI > 25 kg/m^2^ (Grundy et al., 2005) diagnosed prior to initiation of HAART.

Renal impairment was defined as an eGFR ≤90 mL/min/1.73 m^2^ (Consolidated Guidelines on HIV Prevention, Testing, Treatment, Service Delivery and Monitoring: Recommendations for a Public Health Approach, 2021).

Underweight was defined as a BMI < 18.5 kg/m^2^ according to the Department of Health, the Ministry of Public Health, Thailand.

### 2.4 Statistical analysis

Categorical variables were summarized as frequencies and percentages, and analyzed using the chi-square test or Fisher's exact test where appropriate. Continuous variables were summarized as mean ± standard deviation (SD) or median and interquartile range (IQR) values, and compared using the *t*-test or the *Mann–Whitney U test* where appropriate. All variables associated with renal impairment at a *p*-value < 0.1 were included in the logistic regression model. The variables with statistical significance in this model indicated the predictive factors of renal impairment. All the tests for significance were two-sided, and a *p*-value <0.05 was considered statistically significant. Data analysis was performed using IBM® SPSS® Statistics 22.0.

## 3 Results

Among the 315 patients, the distribution of pre-existing metabolic abnormalities was shown in Table 1. The prevalence of elevated low-density lipoprotein (47.3 %) was the highest, followed by hypercholesterolemia (31.4 %). The demographic and baseline clinical characteristics of patients with HIV with and without renal impairment were similar, except that patients with renal impairment were significantly older; their ALT, serum creatinine, proportion of underweight patients, and anti-HCV positivity were significantly higher compared to patients without renal impairment. Conversely, platelet counts and eGFR were significantly lower among those with renal impairment, as shown in Table 2.

**Table 1:** The distribution of pre-existing metabolic abnormalities in patients with HIV and pre-existing metabolic abnormalities in Thailand, *n* = 315 (2015–2018).

Metabolic abnormalities	no. (%)
High LDL	149 (47.3)
Hypercholesterolemia	99 (31.4)
Low HDL	90 (28.6)
Hypertention	90 (28.6)
Hyperglycemia	64 (20.3)
Obesity	51 (16.2)
Diabetes	40 (12.7)

LDL: low-density lipoprotein; HDL: high-density lipoprotein.

**Table 2:** Demographics and baseline clinical characteristics of patients with HIV and pre-existing metabolic abnormalities in Thailand according to renal function after initiating highly active antiretroviral therapy containing tenofovir, *n* = 315 (2015–2018).

Characteristics	Renal impairment# (no.; %)		p-value⁎
	No	yes	
Age (median (IQR)	34 (31–41)	39 (33–46)	<0.01
Male	137 (76.5)	111 (81.60)	0.28
Body mass index (kg/m^2^)	20.94 (18.5–23.3)	21.53 (19.6–23.7)	0.21
Underweight	19 (14.2)	42 (23.9)	0.03
Opportunistic infection on admission	48 (26.8)	43 (1.6)	0.35
Hemoglobin (g/dL; median (IQR)	13.20 (11.5–14.5)	13.30 (11.5–14.4)	0.27
Hematocrit (%; median (IQR)	41.80 (7.2–45.5)	40.90 (36.8–44.7)	0.18
Mean corpuscular volume (fL; median (IQR)	84.40 (77.7)	84.65 (84.4)	0.74
Red blood cell (10^3^/uL; median (IQR)	5.09 (4.4–5.6)	4.91 (4.4–5.3)	0.11
Platelet (10^3^/uL; median (IQR)	254 (218–315)	239 (193–277)	0.01
Alkaline phosphatase (U/L; median (IQR)	67 (43–83)	63 (0–81)	0.42
Alanine transaminase (U/L; median (IQR)	20 (15–30)	23 (17–40)	0.04
Aspartate transaminase (U/L; median (IQR)	23.00 (17.0–33.0)	25.50 (20.0–42.0)	0.08
Creatinine (mg/dL; median (IQR)	0.84 (0.7–0.9)	0.96 (0.9–1.1)	<0.01
eGFR (mL/min; median (IQR)	116 (106–123)	98 (87–108)	<0.01
CD4 (%; median (IQR)	12.00 (4.0–17.0)	11.00 (5.5–18.0)	0.77
Absolute CD4 (cell/mm^3^; median (IQR)	226 (51–358)	186 (95–410)	0.94
HBs Ag positive	9 (5.60)	8 (7.0)	0.64
Anti-HCV positive	2 (1.40)	10 (9.0)	0.01

IQR: interquartile range; eGFR: estimated glomerular filtration rate**;** HBs Ag: Hepatitis B surface antigen; Anti-HCV: Anti-hepatitis C virus.

# Renal impairment was defined as eGFR ≤90 mL/min/1.73m^2^.

⁎ Chi square test/ Mann–Whitney *U* test.

**Fig. 1:**
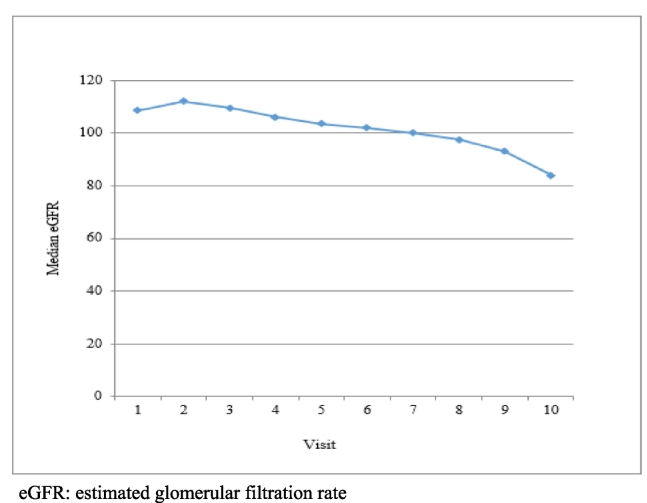
Changes of median estimated glomerular filtration rate of patients with HIV and pre-existing metabolic abnormalities in Thailand after initiating highly active antiretroviral therapy containing tenofovir, n = 315 (2015–2018). eGFR: estimated glomerular filtration rate.

Among the HAART regimens, TDF/FTC/EFV (75.2 %) was the most frequently prescribed, followed by TDF + 3TC + EFV (20.6 %) as shown in Table 3. The eGFR started to decline after 12 weeks of treatment (Fig. 1). The median duration of renal impairment was 43 weeks after starting TDF-based therapy; however, cases were observed as early as 1 week after starting TDF. Multiple logistic regression analysis showed that a BMI < 18.5 kg/m^2^ (odds ratio [OR] = 9.20, 95 % confidence interval [CI]: 1.11, 76.50) and baseline eGFR (OR = 0.92, 95 % CI: 0.89, 0.94) were the predictive factors of renal impairment in patients with HIV and metabolic abnormalities (Table 4).

**Table 3:** Highly active antiretroviral therapy in patients with HIV and pre-existing metabolic abnormalities in Thailand, n = 315 (2015–2018).

ART	no. (%)
TDF/FTC/EFV	237 (75.2)
TDF + 3TC + EFV	65 (20.6)
TDF/FTC + RPV	4 (1.3)
TDF/FTC + LPV/r	3 (0.9)
TDF/FTC + EFV	2 (0.6)
TDF + 3TC + RPV	2 (0.6)
TDF/FTC + NVP	1 (0.3)
TDF + 3TC + LPV/r	1 (0.3)

ART: antiretroviral treatment; / = fixed dose combined pill; TDF: tenofovir, FTC: emtricitabine, EFV: efavirenz, 3TC: lamivudine, RPV: rilpivirine, LPV/r: lopinavir/ritonavir, NVP: nevirapine.

**Table 4:** Multiple logistic regression analysis to investigate the predictive factors of renal impairment in patients with HIV and pre-existing metabolic abnormalities in Thailand, n = 315 (2015–2018).

Predicting factors	Odd ratio (95 %CI) ⁎
BMI <18.5	9.20 (1.11, 76.50)
Hematocrit	1.03 (0.94, 1.14)
Hemoglobin	0.97 (0.84, 1.11)
Baseline eGFR	0.92 (0.89, 0.94)
Duration of TDF treatment	1.00 (0.61, 1.65)
AntiHCV positive	0.45 (0.04, 5.32)
HBsAg positive	0.52 (0.06, 4.66)
CD4 count	1.00 (0.10, 1.00)

eGFR: estimated glomerular filtration rate; TDF: Tenofovir disoproxil fumarate; Anti-HCV: Anti-hepatitis C virus; HBsAg: Hepatitis B surface antigen, CI: confident interval.

⁎ Multiple logistic regressions.

## 4 Discussion

Underweight status and baseline eGFR were identified as predictive factors for renal impairment. After initiating TDF, the eGFR started to decline at 12 weeks in this study, similar to the decline observed at 10 weeks in patients with HIV in South India (Achappa, n.d.). This suggests that, renal function monitoring using eGFR should begin as early as three months after TDF initiation. However, using eGFR to monitor TDF-induced renal impairment may not be feasible. Fortunately, low body weight has been reported as a risk factor for acute kidney injury (Fernandez-Fernandez et al., 2011). Low body weight may serve as a surrogate measure for eGFR decline in TDF-treated patients, since it is associated with eGFR reduction, particularly among individuals with smaller body size, such as Asians (Nishijima et al., 2019). Population pharmacokinetics supports this association: TDF plasma clearance is linked to the body weight-to-serum creatinine ratio, with a lower ratio correlating with a higher tenofovir AUC₀–₂₄ (Jullien et al., 2005). After starting TDF, close monitoring of body weight was suggested in patients with an abnormal baseline eGFR. Each five kg decrement in body weight was significantly associated with TDF-associated renal dysfunction (Nishijima et al., 2011). This approach is simple and amenable to self-monitoring, making it practical for monitoring renal impairment.

Therefore, self-monitoring of body weight may serve as a feasible method for monitoring TDF-associated renal impairment in resource-limited settings. Patients should be encouraged to regularly monitor their body weight and promptly contact healthcare providers if underweight status occurs.

## 5 Strengths and limitations

The strength of this study lies in its contribution as one of the limited number of studies in Southeast Asia that examine the interaction between TDF and pre-existing metabolic conditions. The findings provide practical insights that can improve patient monitoring guidelines, especially for high-risk groups vulnerable to renal impairment. However, the study has some limitations. The dataset covers the period from 2015 to 2018, which may raise concerns about its current applicability. Nevertheless, the study remains relevant for several reasons. TDF continues to be widely used due to its proven efficacy, broad availability, and cost-effectiveness. Furthermore, the long-term renal safety of TDF, particularly among PLHIV with metabolic abnormalities, remains a pressing issue, especially in resource-limited settings. Given the continued use of TDF and the rising incidence of metabolic syndrome among PLHIV, the findings of this study are still highly pertinent.

## 6 Conclusion

In resource-limited countries, healthcare expenditure must be optimized to ensure the delivery of high-quality services. Among PLHIV with pre-existing metabolic abnormalities, underweight and eGFR are the predicting factors of renal impairment. Maintaining normal a BMI and adjusting ART dosages based on renal function are recommended, particularly for patients with abnormal baseline eGFR. Accordingly, the National Health Security Scheme should be revised to better address the specific health care needs of this vulnerable population**.**

## Ethics approval and informed consent statements

This longitudinal study was conducted at a university hospital in Bangkok, Thailand in accordance with the Declaration of Helsinki. Ethics approval was granted by the Rajavithi University Hospital review board on January 16, 2020. The approval number is 63016. Patients registered at HIV clinics from January 2015 until December 2018 were invited to participate in the study and were provided with a written informed consent.

## CRediT authorship contribution statement

**Duangjai Duangrithi:** Writing – review & editing, Writing – original draft, Methodology, Formal analysis, Data curation, Conceptualization. **Kwandaw Silathong:** Writing – review & editing, Writing – original draft, Methodology.

## Funding statement

This research did not receive any specific grant from funding agencies in the public, commercial, or not-for-profit sectors.

## Declaration of competing interest

The authors declare that they have no known competing financial interests or personal relationships that could have appeared to influence the work reported in this paper.

## Data Availability

The data that has been used is confidential.

## References

[bb0005] Achappa B. (2025). Incidence and risk factors for Tenofovir induced nephrotoxicity among patients with HIV on stable combination antiretroviral therapy (c ART) in South India. IJID.

[bb0010] Asirvatham E.S., Ranjan V., Garg C., Sarman C.J., Periasamy M., Vijay Yeldandi V. (2024). A review of tenofovir disoproxil fumarate associated nephrotoxicity among people living with HIV: burden, risk factors and solutions. CEGH.

[bb0015] Bunpeth W., Supasyndh O., Satirapoj B. (2017). Renal injury and dysfunction among HIV positive patients receiving Tenofovir based antiretroviral therapy. J Southeast Asian Med Res..

[bb0020] Calza L. (2012). Renal toxicity associated with antiretroviral therapy. HIV Clin. Trials.

[bb0025] (2021). Consolidated Guidelines on HIV Prevention, Testing, Treatment, Service Delivery and Monitoring: Recommendations for a Public Health Approach.

[bb0030] (2025). Country factsheets, THAILAND 2022, HIV and AIDS Estimates. https://www.unaids.org/en/regionscountries/countries/thailand.

[bb0035] (2017). Department of Disease Control, Thailand National guidelines on HIV/AIDS treatment and prevention. https://www.thaiaidssociety.org/wp-content/uploads/2022/02/Thailand-National-Guidelines-on-HIV-AIDS-Treatment-and-Prevention-2017.pdf.

[bb0040] Division of AIDS and STDs. Annual report (2022). Department of Disease Control, Ministry of Public Health. https://ddc.moph.go.th/das/journal_detail.php?publish=14849.

[bb0045] Duangrithi D., Polsracoo K., Bhuddhataweekul T. (2020). Metabolic abnormalities among HIV-infected patients: the rationale of national health security for people living with HIV. JPTCP.

[bb0050] Ergin H.E., Inga E.E., Maung T.Z., Javed M., Khan S. (2020). HIV, antiretroviral therapy and metabolic alterations: a review. Cureus.

[bb0055] Fernandez-Fernandez B., Montoya-Ferrer A., Sanz A.B., Sanchez-Niño M.D., Izquierdo M.C., Poveda J. (2011). Tenofovir nephrotoxicity: 2011 update. AIDS Res Treat..

[bb0060] Grundy S.M., Cleeman J.I., Daniels S.R., Donato K.A., Eckel R.H., Franklin B.A. (2005). Diagnosis and Management of the Metabolic Syndrome. Circulation.

[bb0065] (2025). HIV and AIDS, Fact sheet. https://www.who.int/news-room/fact-sheets/detail/hiv-aids.

[bb0070] Jantarapakde J., Phanuphak N., Chaturawit C., Pengnonyang S., Mathajittiphan P., Takamtha P., Dungjun N. (2014). Prevalence of metabolic syndrome among antiretroviral-naive and antiretroviral-experienced HIV-1 infected Thai adults. AIDS Patient Care STDS.

[bb0075] Jullien V., Tréluyer J.M., Rey E., Jaffray P., Krivine A., Moachon L. (2005). Population pharmacokinetics of tenofovir in human immunodeficiency virus-infected patients taking highly active antiretroviral therapy. Antimicrob. Agents Chemother..

[bb0080] Nishijima T., Komatsu H., Gatanaga H., Aoki T., Watanabe K., Kinai E. (2011). Impact of small body weight on tenofovir-associated renal dysfunction in HIV-infected patients: a retrospective cohort study of Japanese patients. PloS One.

[bb0085] Nishijima T., Gatanaga H., Oka S. (2019). Tenofovir nephrotoxicity among Asians living with HIV: review of the literature. Glob Health Med..

[bb0090] Phuphuakrat A., Pasomsub E., Chantratita W., Mahasirimongkol S., Disthabanchong S., Sungkanuparph S. (2022). Risk factors of renal tubular dysfunction in thai people living with hiv receiving tenofovir disoproxil fumarate. JIAPAC.

[bb0095] Pichpattana M., Phiboonbanakit D., Trakulhun K., Supasyndh O. (2016). Incidence of Tenofovir Disoproxil fumarate induced proximal Tubulopathy in HIV-infected patients. J Infect Dis Antimicrob Agents..

[bb0100] Ruxrungtham, K., Chokephaibulkit, K., Chetchotisakd, P., Chariyalertsak, S., Kiertburanakul, S., Putacharoen, O., et al., 2022. Thailand National Guidelines on HIV/AIDS Treatment and Prevention 2021/2022. Nonthaburi, Division of AIDS and STIs, Department of Disease Control. https://www.thaiaidssociety.org/wp-content/uploads/2023/03/HIV-AIDS-Guideline-2564_2565_ED2.pdf. (Accessed 20 February 2024).

[bb0105] Srisopa S., Kornjirakasemsan A., Treebupachatsakul P., Sonthisombat P. (2023). Incidence and risk factors of tenofovir disoproxil fumarate induced nephrotoxicity and renal function recovery, a hospital case-control study. Infect Chemother..

[bb0110] Suwan D., Kornjirakasemsarn A. (2020). Incidence and factors associated with Tenofovir-induced nephrotoxicity in HIV infected patients in Nakornping hospital. J. Nakornping Hosp..

